# Case Report: Relapse of SLE associated with EBV infection in a patient after CAR-T cell therapy: a clinical case

**DOI:** 10.3389/fimmu.2026.1956166

**Published:** 2026-09-30

**Authors:** Ping Zeng, Qi Ren, Xianfei Gao, Wei Xian, Pui Y. Lee, Feng Li, Caihong Xu, Mingqi Zhao, Wenhao Zhou

**Affiliations:** 1 Zunyi Medical University, Zunyi, Guizhou, China; 2 Department of Pediatric, Allergy Immune & Rheumatology, Guangzhou Women and Children’s Medical Center, Guangzhou Medical University Guangdong Provincial Clinical Research Center for Child Health, Guangzhou, China; 3 Division of Immunology, Harvard Medical School, Boston, MA, United States

**Keywords:** CAR- T cell therapy, chronic active EBV disease, Epstein–Barr virus, pediatric lupus, reactivation, systemic lupus erythematosus

## Abstract

We present the case of a 13-year-old boy with refractory systemic lupus erythematosus (SLE) who achieved drug-free remission after anti-CD19 CAR-T cell therapy, but subsequently experienced Epstein–Barr virus (EBV) reactivation on day 142 after CAR T-cell infusion and peak EBV load was 24,400 IU/mL with a severe drop in platelets to 8×10^9^/L, which leading to relapse of SLE and manifestations of chronic active EBV disease (CAEBVD). The whole exogenous gene testing found X chromosome Xp22.33q28 region (chrX: 200855-155239820) a duplicate of about 155.04Mbp,which is associated with the development of Klinefelter syndrome (KS).The temporal association between EBV resurgence and SLE flare suggests that CAR-T-induced immunosuppression with some genetic background may permit EBV reactivation, which can be associated with the return of autoimmunity. This case highlights the need for vigilant monitoring for opportunistic infections and genetic background, particularly EBV, after CAR-T therapy for autoimmune diseases, as they may contribute to disease relapse.

## Introduction

Systemic lupus Erythematosus (SLE) is a chronic multi-system autoimmune disease characterized by autoantibodies against nuclear antigens, immune complex deposition and chronic inflammation of classic target organs such as skin, joints and kidneys (1). About 3.4 million people worldwide have been diagnosed with SLE. The overall incidence of SLE is 0.3-23.2 cases/100,000 years, and the incidence ratio between women and men is about 8:1. At present, the exact etiology of SLE is not clear, but genetic and environmental factors (such as sunlight), changes in hormone levels, infections and certain drugs may all play a role in the occurrence of SLE disease (2).

EBV infection is highly prevalent in the general population. Greater than 95% of adults have experienced EBV infection and many have no specific symptoms during acute infection as manifestations of infectious mononucleosis are less common in adults (3). The virus is also associated with the development of nasopharyngeal cancer, lymphoid hyperplasia, and a number of autoimmune diseases (4, 5). Accumulating evidence points to EBV as a potential co-factor in the development of SLE (6).

EBV infection plays an important role in the onset of SLE in children. Current clinical evidence points more to its involvement in the start-up phase of the disease. The rate of recent EBV infection is higher in children with SLE, and the titer of anti-EBV antibodies (such as EBV EA-D IgG) is significantly higher than that of health controls (7, 8). It is suggested that EBV infection may be related to the onset of SLE. The EBV DNA load in the peripheral blood of pediatric SLE patients is increased significantly; abnormal expression of genes associated with the incubation period (such as LMP1) and cell lysis are indicative of active EBV infection or reactivation (9, 10). However, there is no clear correlation between EBV infection and the severity and activity of SLE disease, indicating that EBV may mainly work in the early stage of triggering the disease, rather than determining the severity of disease complications (11–13).

CAR-T therapy (chimeric antigen receptor T cell therapy) transforms the patient’s own T cells through genetic engineering so that it can accurately identify and destroy specific targets. CAR-T for SLE mainly targets CD19 (or BCMA) on the surface of B cells. After transfusion, CAR-T cells will remove almost all CD19+ B cells. At present, CAR-T therapy targeting CD19 has been used in many centers around the world to treat intractable and severe SLE patients (including child and adolescents) (14–17). Theoretically, this process also removes the EBV-rich B cell reservoir in SLE patients, which may break the vicious circle of the disease at its root. Clinical results show that many treated patients achieve rapid and deep disease relief after B cells are removed, and all immunosuppressants and hormones can even be discontinued for years (18–22). With the reconstruction of B cells, the immune system seems to be “reset”. The main short-term risks are cytokine release syndrome (CRS) and increased risk of infection (due to B cell clearance). Whether EBV infection after CAR-T treatment will cause SLE reactivation has not been mentioned.

## Case presentation

We investigated a 13-year-old Chinese boy who received anti-CD19 CAR-T cell therapy for his intractable SLE (MR-44-25-033303). Five years ago (June 2019), the boy first presented to the rheumatology and immunology department with a complaint of fever, neck swelling for ten days. CT scan showed hepatosplenomegaly, pelvic fluid accumulation and ground-glass opacity in both lungs. Laboratory tests revealed WBC9.9×10^9^/L, PLT5×10^9^/L, Hb 42g/L, antinuclear antibody titer (ELISA) 179.29 IU/L, anti-dsDNA titer (ELISA) 300.00 IU/L, C4 <0.02 g/L, C3 0.13 g/L, serum ferritin 339.00 ng/mL, EB virus detection (fluorescence PCR) ±, EB virus DNA quantitative detection (fluorescence PCR) 5.91×10³ copies/ml. After further examination of bone marrow smear and lymph node biopsy, lymphoma was excluded. A diagnosis of SLE and EB virus infection was considered. SLEDAI 2000 was 12. He was treated with methylprednisolone pulse therapy and belimumab infusion, and hydroxychloroquine,Valacyclovir orally. The condition improved and EBV detection turned negative (<50/ml). Two weeks later, a second application of belimumab caused a generalized rash, which was considered an allergic reaction, and the drug was discontinued. Monthly methylprednisolone pulse therapy and mycophenolate mofetil were applied; the platelet count gradually stabilized and was maintained at 100–250×10^9^/L. From April 2020 to October 2024, the boy came to the clinic every 3 months; the dose of prednisone was gradually reduced from 60 mg per day to 10 mg per day. Annual quantitative tests of EB virus showed negative. But the boy was hospitalized due to recurrent generalized ecchymosis in December 2024. The platelet number dropped to a minimum of 3×10^9^/L with C3 0.31,C4 0.03 and ANA 56,DsDNA 271. EBV testing was negative. IVIG was given and methylprednisolone IV infusion was performed, followed by cyclosporine substituted for mycophenolate mofetil and intravenous rituximab to achieve better disease control and help restoring the low platelet count. Unexpectedly, the third application of rituximab resulted in generalized rash, which was considered as an allergic reaction, and the drug was discontinued.

The boy joined the CAR-T clinical trial and was assigned to the low-dose (0.5×10^5^/kg) group. The home dose of glucocorticoids and other immunosuppressants was stopped 7 days before infusion. Lymphodepletion therapy used fludarabine phosphate(25 mg/m2/day for 3 days) and cyclophosphamide((500 mg/m2/day for 3 days). On 7th May, 2025(day0), the infusion of anti-CD19 CAR-T cells was performed. No adverse reactions (such as cytokines release syndrome) were observed. Four months follow-up showed a SLEDAI score of 4, indicative of low disease activity. On day 142 after CAR T-cell infusion, the boy suffered from fever and thrombocytopenia again. Further laboratory tests revealed WBC 3.2×10^9^6,Hb 89g/L,Plt 7×10^9^.C3 0.19,C4 <0.02,dsDNA 416IU/mL,ANA 8.05 IU/mL, The quantitative EB detection result in plasma was 24,400 IU/mL. In the next 2 weeks, kidney damage and exudation appeared gradually. Urine protein quantification test was 3.78g/24h. Urinary red blood cell count was4365/ul. The X-ray shows that both lungs are blurred, and a small amount of pleural fluid accumulates on the right side. Bone marrow examination showed erythron、granulocyte and megakaryocyte proliferation. Evidence of developmental impairment can be seen in megakaryotic cells and platelets (**Figure 1**). The diagnosis of SLE and EB virus infection was considered again. On day 153, detection of lymphocyte subgroups infected with EB virus revealed that CD8+, CD19+, and NK cells were infected with EBV while CD4+ cells were spared (**Table 1**). On day 165, we used Flow-FISH technology to identify the types of myelocytic and lymphocytic cells infected by EBV and distinguish between B cells, T cells, and NK cells. The results (**Table 2**) showed an imbalance of lymphocyte subsets with decreased total T cells (especially CD4+) and decreased NK cells. EBV+ cells were CD3+CD4+CD8+ T cells, B cells, and NK/NKT cells, with B cells accounting for the highest proportion, followed by NK/NKT cells. High PD-L1 expression in EBER-positive cells of total T cells、CD4^+^ and CD8^+^ T cells suggests possible immune escape (20–22). The overall peripheral blood microenvironment showed no evidence of immunosuppression, but T-cell exhaustion was present. All of these results indicated that the boy may have acute severe EBV infection that coincided with a flare of lupus disease.

**Figure 1:**
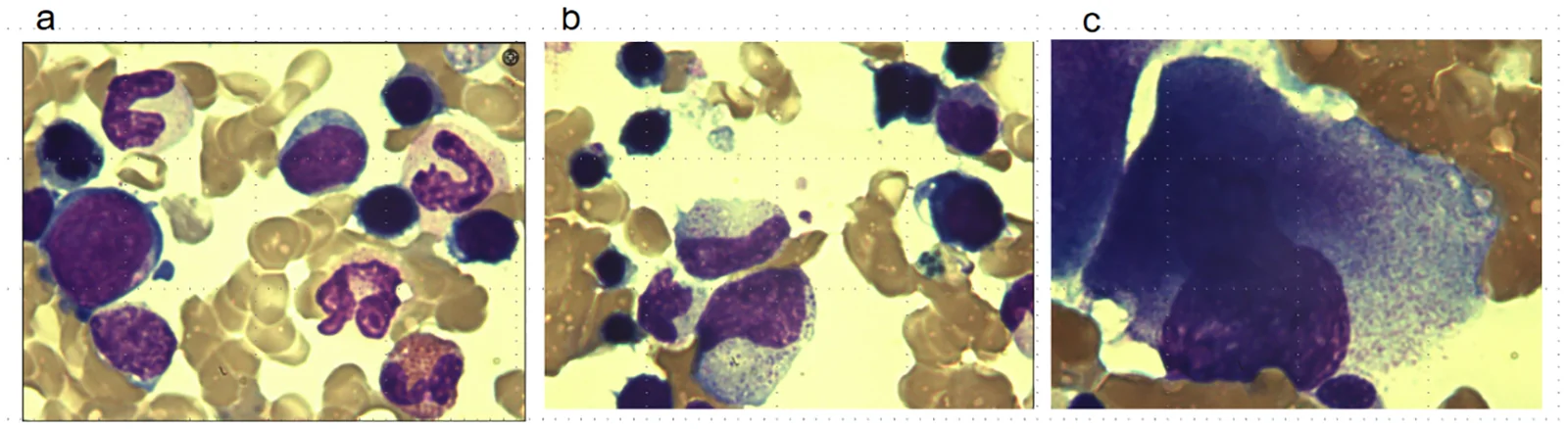
Day 141 examination of bone marrow* * **(a, b)** staining of bone marrow aspirate illustrating normal presence of erythrons, granulocytes and megakaryocytes, and a paucity of platelets. **(c)** Mature disordered megakaryocytes, granular, platelets are rare.

**Table 1:** Detection of lymphocyte subgroups infected with EBV(Day 153).

Lymphocyte subgroups	Before sorting	After the selection	Number of sorted cells	Purity (%)	EBVDNA test results (Copies/millions of cell)
CD3+CD4+T	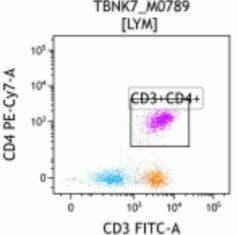	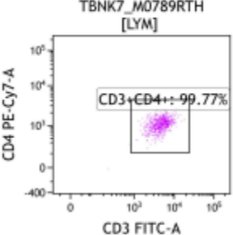	487735	99.77	None
CD3+CD8+T	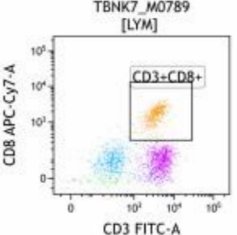	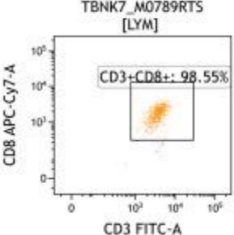	251164	98.55	5.22 × 10^^^²
CD3−CD19−B	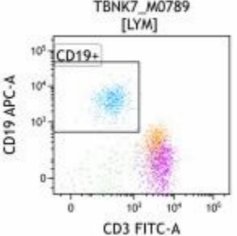	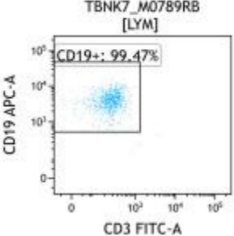	393067	99.47	6.20 × 10^^^³
CD56+ NK/NKT	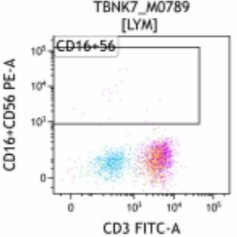	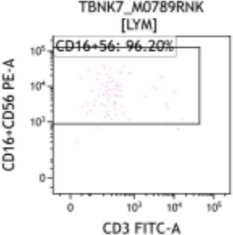	15499	96.20	1.33 × 10^^^³

Method: After freshly prepared cells were washed once in phosphate buffered saline (PBS) (Beijing Solarbio Science & Technology Co, Ltd, no. P1020), all samples were sorted on the FACSMel-ody (BD). Cells from PB samples were stained with anti-CD3 anti-CD4, anti-CD8,anti-CD19, and anti-CD56 antibodies (**Table 1**, available on the Blood website) and subsequent sorting for the CD3’CD4*T cells,CD3’CD8* Tcells,CD3 CD19* B cells, and CD56* NK/NKT cells. Cells from BM aspirates were isolated by staining with anti-CD34 antibod) (**Table 1**) and subsequent sorting for the CD34cells. EBV-DNA copies were quantified in BM aspirates, PB, and sorted cell populations using a commercial PCR diagnostic kit for EBV (ShanghaiZ Bio-Tech Co, Ltd, no.P20220201) according to the manufacturer’s recommendations.

**Table 2:** Flow-FISH identifies EBV infection in the subgroups of myelocytic and lymphocytic cells (Day 165).

Cell subgroup	Cell Proportion(%)	EBER+ cells (/1.00E+06 cells)	The proportion of PD-L1+ cells (%) in EBER+ cells
T3(CD3+)	44.05%	9901	33.33%
T4(CD3+CD4+)	19.05%	10622	42.86%
T8(CD3+CD8+)	18.21%	9615	50%
NK(CD3-CD16&56+)	2.91%	180952	15.79%
B(CD3-CD19+)	16.25%	16043	11.11%
Monocytes(CD14+)	5.64%	28455	14.29%
Neutrophils(CD15+)	53.99%	44816	11.29%

EBER, Epstein-Barr Virus-Encoded RNA;PD-L1,rogrammed Cell Death Ligand 1.

* The Flow-FISH method :1. Isolate peripheral blood single nuclear cells;2. Fluorescent antibody labeling for specific cell surface markers of different cell subgroups of gonorrhea and medullary system. 3. Fluorescent probe FISH hybridization for EBV-specific nucleic acid marker EBER; 4. Use a flow cytometer to detect fluorescent antibodies and fluorescent probe markers of different cell subgroups at the same time.

During the observation and follow-up periods after CAR-T infusion, the number of CAR T-cells significantly increased after the administration, peaking on days 7–10 and then undergoing a typical contractions (**Figure 2d**). CD19+ cell depletion was observed on day 7, and the cells were reconstituted by day 60 (**Figure 2a**). Laboratory monitoring revealed a pivotal role of CART-T dynamics. The peak of CART cell amplification was day 10 and then declined to the trough on the day 40 (**Figure 2d**). Correspondingly, the platelet count was low (6–10×10^9^/L) initially, improved to 153×10^9^/L by day 40 and maintained by day 109 (**Figure 2e**), which demonstrated the effectiveness of CART therapy. During the period of first 30 days of CART therapy, peripheral blood white blood cell counts were less than 3×10^9^/L (**Figure 2f**), with a lymphocyte count less than 0.5 × 10^9^/L. However, a subsequent and dramatic surge in EB DNA load to 24,400 IU/mL by day 142 was accompanied by a severe drop in platelets to 8×10^9^/L (**Figures 2c, e**), highlighting the temporal association between high levels of viral replication and disease relapse. Concurrently, CD19+ cells were effectively depleted initially (5.3% at day 7) but reconstituted by day 40 (51.2%) and persisted at 36% on day 142 despite the high EBV load. Peripheral blood white blood cell 、neutrophil、lymphocyte 、CD8+、CD4+ and CD3+ cell counts fluctuated but showed an overall increasing trend (**Figure 2a, f**). Complement levels C3 and C4 improved gradually after CAR-T cell infusion and were nearly normal on day 60 before dropping precipitously (**Figure 2b**). CAR T-cell therapy was well-tolerated, but the condition seem to have a striking inverse correlation with EBV. Plasma EBV DNA was 45 IU/mL at day -5, became undetectable (0 IU/mL) on day -1 and day 7, and showed a transient, low-level rebound to 98 IU/mL on day 3 and 422 IU/mL on day 42 (**Figure 2c**). These observations suggest that EBV has been dormant in the body, reaching undetectable levels by PCR testing as CAR-T cells cleared B cells. The sharp increase in the number of viruses may be associated with the EBV reactivation or reinfection.

**Figure 2:**
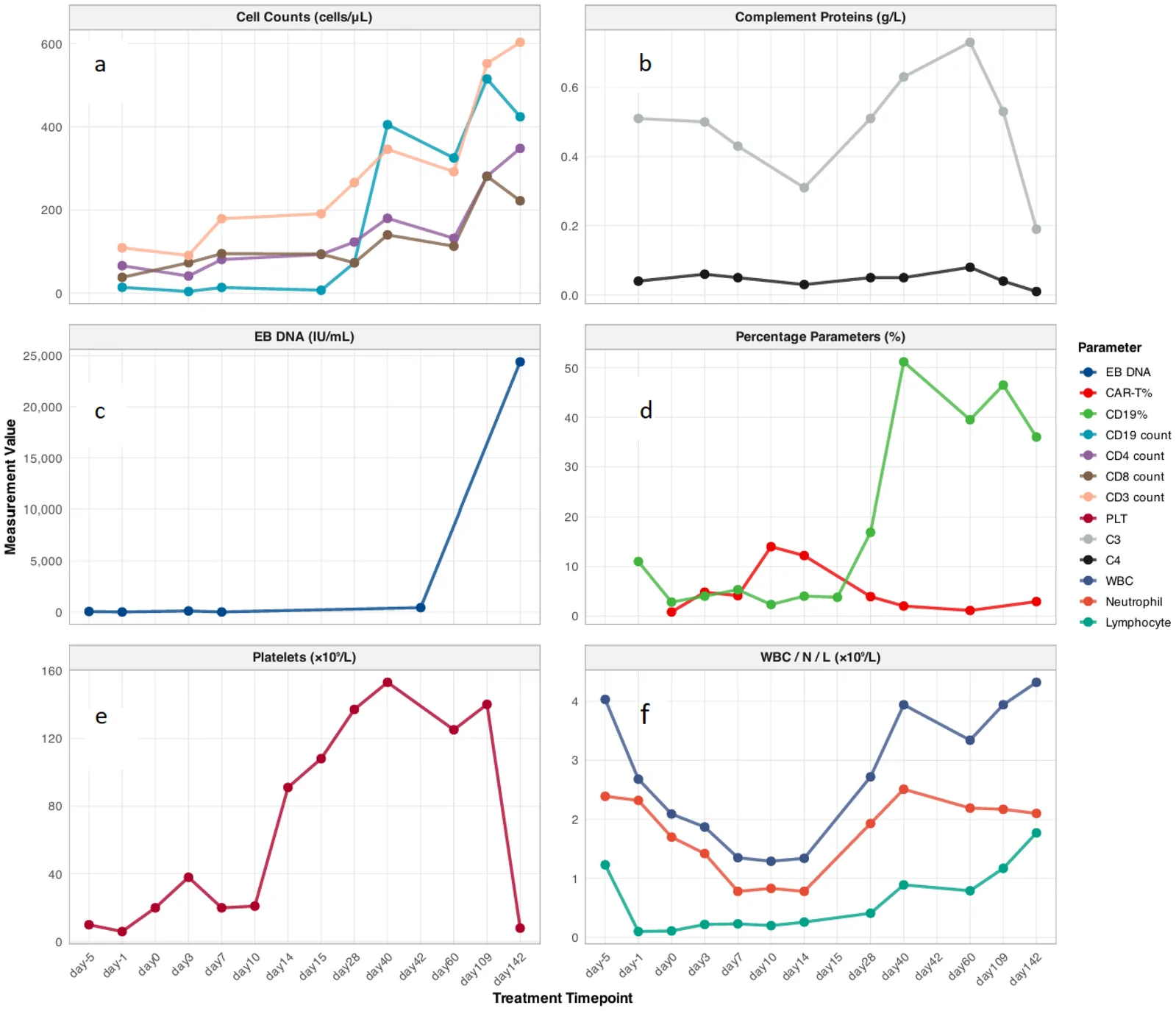
During CART therapy,measurement of EB virus, CAR T cells, lymphocyte subsets, platelet count, and complement levels, peripheral blood white blood cell counts.* *EBV detection method: Blood samples were collected, and peripheral blood mononuclear cells were isolated within 2 hours. Then the cells were treated with TRIzol (99088101, Invitrogen USA) and stored in -80°C until DNA isolation. EBV nucleic acid detection kit (PCR-fluorescent probe method) (20173403080, Huaruian Biology, Guangzhou, China) was used to quantify EBV DNA. Day 0 was the date of CART-cell infusion.

There are no family members with a similar condition. In order to further explore the relationship among genetic background、EBV infection and systemic lupus erythematosus, we performed whole exogenous gene testing for the boy. We found the subject’s X chromosome Xp22.33q28 region (chrX: 200855-155239820) a duplicate of about 155.04Mbp.The region includes a total of 1886 genes, of which 841 are protein-encoding genes. The Xp22.33q28 region of X chromosome of the subject covers the entire length of X chromosome. This duplicate variant was not included in the DGV database (**Figure 3**). X chromosome duplication in men is associated with the development of Klinefelter syndrome (KS).Klinefelter Syndrome is a disease of sex chromosome variants, the most common karyotype is 47, XXY, and has been reported in multiple patients (23, 24). The variant is classified as a pathogenic variant according to ACMG guidelines. Klinefelter syndrome is a common sex chromosome abnormality and the most common disease that causes male sexual dysfunction. Increased risk of diseases such as systemic lupus erythematosus, rheumatoid arthritis and Sjogren’s syndrome. There is no direct causal relationship between Klinefelter syndrome and Epstein-Barr virus (EBV) infection according to the current medical literature.

**Figure 3:**
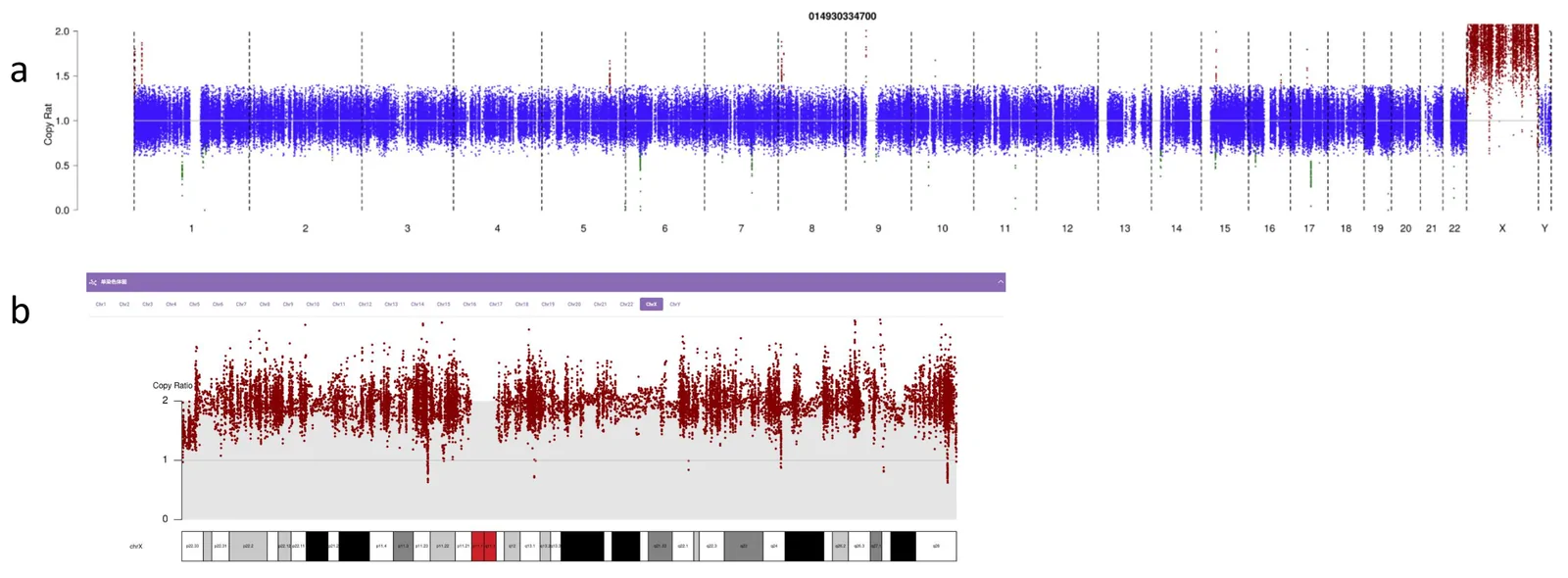
The X chromosome region Xp22.33q28 of the subject* * a.Whole-exome high-throughput sequencing technology to sequence genomic DNA, a repeat region of approximately 155.04 Mbp was identified in the Xp22.33q28 region of the X chromosome (chrX:200855–155239820) in the subject. **(b)** This region encompasses a total of 1,886 genes, of which 841 are protein-coding genes.

## Discussion

CAR-T therapy has been a breakthrough in the treatment of refractory systemic lupus erythematosus with remarkable effects. Its impact is mediated by near complete removal of B cells that produce pathogenic autoantibodies in the body. According to the early data of many clinical studies at present, CAR-T therapy has shown long-term and stable drug-free remission for the treatment of systemic lupus erythematosus, with low overall recurrence rate (19, 25–27). In existing studies, disease recurrence is characterized by reappearance of pathogenic memory B cells or plasma cells, leading to continued production of autoantibodies. Incomplete clearance of target B lymphocytes may be due to the lack of CD19 antigens in some subsets (such as some plasma cells), thus avoiding the attack of CAR-T cells (28, 29).

The recurrence rate after CAR-T treatment varies significantly depending on the underlying disease. Recurrence is very common in hematological malignancies, while low response rate and complex recurrence patterns are more common in solid tumors (30–33). Recurrence of autoimmune diseases has been observed but the frequency has not been established (34, 35). Disease relapse after CAR-T treatment.is a complex problem with multiple factors. According to the experience in the field of hematological malignancies, the causes of recurrence are summarized into several aspects including target cells, CAR cell characteristics, and immune suppression conditions. Infection is very common and the incidence rate ranges from 17% to 80% after CAR-T therapy (36–40). Notably, infections such as viruses, bacteria, parasites and fungi play an important role in the development of autoimmune diseases. In particular, it is reported that the virus infection, especially EBV, is involved in the development of systemic lupus erythematosus. Viral infections have been implicated in the pathogenesis of autoimmune diseases through a variety of mechanisms, including structural or functional molecular mimicry/cross-reactivity, innate immune activation by IFN production, epigenetic factors, superantigen generation, bystander activation, regulation of apoptosis and clearance, and epitope spreading (41). Reassuringly, the incidence of severe infection after CAR-T cell treatment in patients with autoimmune diseases is very low.

Our clinical trial(MR-44-25-033303) aims to explore the efficacy and safety of different doses of CAR-19 on systemic lupus erythematosus in children. The boy was randomized to the low-dose group(0.5×105/kg). There were no complications such as CRS that occurred after CAR-T cell infusion. Drug-free remission was achieved within 4 months. EB virus infection was observed after 4 months, which progressed to chronic active EB virus infection concomitant to a return of SLE activity. We detected lymphocyte subgroups infected with EBV on day 153 and found CD8+、CD19+、NK cells has different possibility of active EBV infection except CD4+ (**Table 1**) .But on day 165, NK cells have a high possibility of active EBV infection (180,952 EBER-positive cells detected per 1,000,000 cells). CD19+ cells have a relatively low but noticeable possibility of active EBV infection (16,043 EBER-positive cells detected per 1,000,000 cells). CD4 T cells may have active EBV infection (10,622 EBER-positive cells detected per 1,000,000 cells). EBER-positive cells in total T cells, CD4 T cells, and CD8 T cells show high PD-L1 expression, suggesting possible immune evasion. EBV was spreading rapidly. There may be EBV active infection in multiple cell lineages: NK cells, B cells, CD4 T cells. Reduced proportion of CD4 T cells means T cell immune abnormalities、T cell immune evasion and exhaustion. Some studies found 30% or more of the EBER-positive cells express PD-L1 indicated this cell subgroup might have the potential to escape the immune system (42–44). EBV-infected cells differentiate into memory B cells, which persist throughout their lives and may be activated again under certain conditions. The triggers of reactivation are not completely clear and may be related to immune status, such as stress, fatigue, other infections, immunosuppression and some genetic background. Long-term immunosuppression (especially in the setting of impaired B cell and T cell function) caused by CAR-T treatment is likely a key factor for the re-infection or activation of EBV. Then, genetic background is an important cause of recurrence of lupus erythematosus.

The relationship between CAR-T treatment and EBV infection is bidirectional: on one hand, CAR-T can be designed as an active weapon to target the removal of EBV infected cells; on the other hand, EBV infection may also occur as a complication after CAR-T treatment, although it is a more common complications after solid organ transplantation or allogeneic hematopoietic stem-cell transplantation(HSCT) (45, 46). Our case was first diagnosed of SLE with EBV infection. EBV infection is not the only cause of SLE, but it is likely to play a key “trigger” role in individuals with genetic predisposition. Current studies on Klinefelter syndrome (KS, 47, XXY) and EBV-related diseases are very limited, and there is no recognized direct, universal causal relationship between them. Patients with KS are at higher risk of lymphoma, and EBV is a known causal factor for some lymphomas (47). Both KS and EBV are risk factors for SLE. KS may provide a “susceptible” background for EBV to trigger SLE by affecting sex hormones and the immune system, which is mainly reflected in EBV-related lymphoma, autoimmune diseases, and the superposition of genetic background. The boy’s inheritance background and EBV infection were important causes of SLE. After CAR-19 therapy and complete remission for 4 months, the EBV load increased sharply, and B cells were abnormally proliferative in association with a return of lupus manifestations such as thrombocytopenia and kidney damage (proteinuria, hematuria). Subsequently, EBV quickly infected CD3+, CD4+, CD8+, NK cells, B cells, monocytes, and neutrophils. PD-L1 is a transmembrane protein expressed on the surface of various cells. Its primary function is to bind to the PD-1 receptor on T cells, which transmits an inhibitory signal to T cells, suppressing their proliferation, activation, and cytotoxic activity. EBV strongly upregulates PD-L1 on the surface of infected cells through multiple products it encodes, at various levels. High levels of PD-L1 can effectively inhibit the antiviral activity of surrounding T cells and NK cells, creating conditions for persistent virus infection and disease progression. Surviving PD-L1 infected T cells secrete inflammatory factors such as IFN, which strongly drive B cells to differentiate into plasmablasts and produce autoantibodies (48, 49). Extensive T-cell EBV infection and high PD-L1 expression suggest chronic active EBV infection (50).

The boy is being evaluated for stem cell transplantation. Currently, the only recognized curative treatment for CAEBV is allogeneic hematopoietic stem cell transplantation (allo-HSCT). Data indicate that child and adolescent patients have relatively favorable prognoses, with 3-year overall survival (OS) rates ranging from 60% to 87% (51). The success rate is associated with factors such as whether the patient had hemophagocytic lymphohistiocytosis (HLH) prior to transplantation, pre-transplant EBV-DNA viral load, and pre-transplant liver function indicators (51–56). Given the boy’s high EBV viral load and active systemic lupus erythematosus (SLE) condition, he is receiving anti-EBV and immunosuppressive treatment. The condition is gradually stabilizing and being prepared for transplantation.

In summary, we reported a 13 years old boy with Klinefelter syndrome who received CAR-19 therapy and obtained drug-free remission for 4 months. However, EBV infection was found around the time of lupus disease flare. This case illustrates the need for assessment of genetic background and careful monitoring of opportunistic infection such as EBV after successful treatment with CAR-T cells. Due to the rarity of the disease, no similar cases were found in this study for comparative analysis, so the conclusions drawn may have certain limitations.

## Data Availability

The raw data supporting the conclusions of this article will be made available by the authors, without undue reservation.
